# Blue Toe Syndrome and Acute Kidney Injury Following Transcatheter Aortic Valve Replacement: A Biopsy-Proven Case of Cholesterol Embolization Syndrome

**DOI:** 10.7759/cureus.117137

**Published:** 2026-09-29

**Authors:** Varun Mehta, Nelson Reyes Perez, Eleana Estrella, Shivam Joshi

**Affiliations:** 1 Department of Internal Medicine, University of Central Florida, Orlando, USA; 2 Department of Medicine, Division of Nephrology, Orlando VA (Veterans Affairs) Healthcare System, Orlando, USA

**Keywords:** acute kidney injury, atheroembolic renal disease, blue toe syndrome, cholesterol embolization syndrome, shaggy aorta, transcatheter aortic valve replacement

## Abstract

Cholesterol embolization syndrome (CES) is an underrecognized complication of arterial instrumentation caused by embolization of atheromatous debris into small- and medium-sized arteries. Acute kidney injury (AKI) is well described after transcatheter aortic valve replacement (TAVR), but biopsy-proven CES after TAVR is rarely reported. Its delayed presentation may be mistaken for contrast-associated nephropathy, rapidly progressive glomerulonephritis, or systemic vasculitis. A 77-year-old man with chronic kidney disease, type 2 diabetes, hypertension, and peripheral artery disease underwent transfemoral TAVR for severe aortic stenosis. Preprocedural computed tomography angiography showed a "shaggy aorta." One month later, he developed intermittent, painless violaceous discoloration of the left first through third toes. Approximately 2.5 months after TAVR, serum creatinine had risen from a baseline of 1.2-1.5 mg/dL to 4.24 mg/dL, prompting admission. Microscopic hematuria, mild eosinophilia (0.64 ×10³/µL), elevated inflammatory markers, and dysmorphic erythrocytes raised concern for glomerulonephritis and vasculitis, and empiric intravenous methylprednisolone was given while diagnostic evaluation proceeded. Kidney biopsy demonstrated atheroembolic cholesterol clefts in intrarenal arteries with ischemic changes, without glomerulonephritis or immune complex deposition. Preserved pedal pulses with an ankle-brachial index of 1.21 and toe-brachial index of 0.98 supported distal microembolization rather than large-vessel occlusion. Corticosteroids were stopped, nephrotoxins withheld, aspirin resumed, and high-intensity statin therapy was initiated; creatinine improved to approximately 3.1 mg/dL by discharge. CES should remain in the differential for progressive AKI with blue toe syndrome after TAVR, even when presentation is delayed, and initial findings suggest immune-mediated disease.

## Introduction

Cholesterol embolization syndrome (CES), also termed atheroembolic disease, is a multisystem disorder caused by embolization of cholesterol crystals from ulcerated atherosclerotic plaques into small- and medium-sized arteries, typically 100-200 µm in diameter, where they produce a microcrystalline angiitis: neutrophil and eosinophil infiltration of the affected arteriole, followed by mononuclear infiltration, giant-cell formation, thrombosis, endothelial proliferation, and intimal fibrosis, culminating in arterial obstruction [1,2]. Because the crystals are insoluble and cannot be cleared by phagocytosis, they persist indefinitely [2]. This two-stage mechanism, including immediate mechanical occlusion followed by a delayed inflammatory endarteritis, helps explain the characteristically subacute, stepwise clinical course [1]. Throughout this report, CES denotes the systemic syndrome and atheroembolic renal disease as its renal manifestation.

Although spontaneous cases occur, most episodes follow vascular instrumentation, and anticoagulant or thrombolytic therapy is an additional precipitant [1,3]. In a prospective study of consecutive patients undergoing cardiac catheterization, clinically apparent CES occurred in 1.4%, and postprocedural eosinophilia (>500/µL) was significantly more frequent in affected patients, with eosinophil counts rising from 220 to 535 cells/µL specifically in those who developed renal dysfunction [4]. The kidneys, skin, gastrointestinal tract, retina, and central nervous system are most often affected [1]. Renal involvement typically manifests as subacute kidney injury, while livedo reticularis and blue toe syndrome are the classic cutaneous clues, though skin findings are present in only a subset of patients [1,5].

Diagnosis is frequently delayed because the presentation overlaps with more common conditions. The typical patient is a man older than 60 with known atherosclerosis presenting with the triad of a precipitating event, acute or subacute renal failure, and characteristic skin findings, with eosinophilia raising further suspicion [1,3]. Nevertheless, subacute renal dysfunction with eosinophilia, elevated inflammatory markers, hematuria, and constitutional symptoms may initially suggest systemic vasculitis, rapidly progressive glomerulonephritis, thrombotic microangiopathy, or contrast-associated nephropathy [1,5]. Histopathologic demonstration of biconvex, needle-shaped, empty cholesterol clefts ("ghost cells") within arcuate and interlobular arteries remains the diagnostic reference standard [1,2].

Transcatheter aortic valve replacement (TAVR) is now an established therapy for severe symptomatic aortic stenosis across the surgical risk spectrum. Acute kidney injury (AKI) after TAVR is common and is reported in approximately 4.7% in a nationwide cohort using two-sample creatinine ascertainment [6], and can be as high as 17% when broader criteria and longer ascertainment windows are applied [7]. It carries substantial prognostic weight, with a nationwide Danish cohort demonstrating a 7.2-fold higher one-year rate of dialysis and a 2.4-fold higher rate of death among patients with post-TAVR AKI [6]. Embolization of calcific and atheromatous debris during TAVR is well documented: cerebral embolic protection filters capture debris in essentially all patients, comprising acute thrombus, arterial wall and valve tissue, calcification, and foreign material [8-10]. Attention, however, has focused almost exclusively on cerebral embolization; the risk of systemic (non-cerebral) embolization after TAVR is acknowledged but essentially unquantified in clinical practice [9]. Patients with extensive aortic atheroma, particularly a "shaggy" aorta, may be at heightened risk of distal atheroembolism during catheter and delivery-sheath manipulation [11,12].

We report a biopsy-proven case of CES presenting several weeks after TAVR in a patient with diffuse thoracoabdominal atherosclerosis, in whom progressive AKI, blue toe syndrome, eosinophilia, and elevated inflammatory markers initially suggested systemic vasculitis.

## Case presentation

A 77-year-old man underwent elective transfemoral TAVR with a 34-mm self-expanding valve for severe symptomatic aortic stenosis approximately 2.5 months before the index admission. His history included hypertension, type 2 diabetes mellitus, chronic kidney disease (baseline serum creatinine 1.2-1.5 mg/dL), chronic obstructive pulmonary disease, peripheral artery disease with prior right above-knee amputation, abdominal aortic aneurysm, nephrolithiasis, and benign prostatic hyperplasia. Outpatient medications included aspirin, losartan, and empagliflozin.

Preprocedural computed tomography (CT) angiography for TAVR planning demonstrated extensive atherosclerotic disease of the thoracic and abdominal aorta with bulky endoluminal plaque and a "shaggy" appearance (Figure 1). The procedure itself was uncomplicated, and he was discharged on aspirin without therapeutic anticoagulation. Serum creatinine was 1.4 mg/dL on the day after TAVR, remaining within his preprocedural baseline range of 1.2-1.5 mg/dL. His early postprocedural course was notable for transient heart failure with subsequent recovery of left ventricular systolic function.

**Figure 1:**
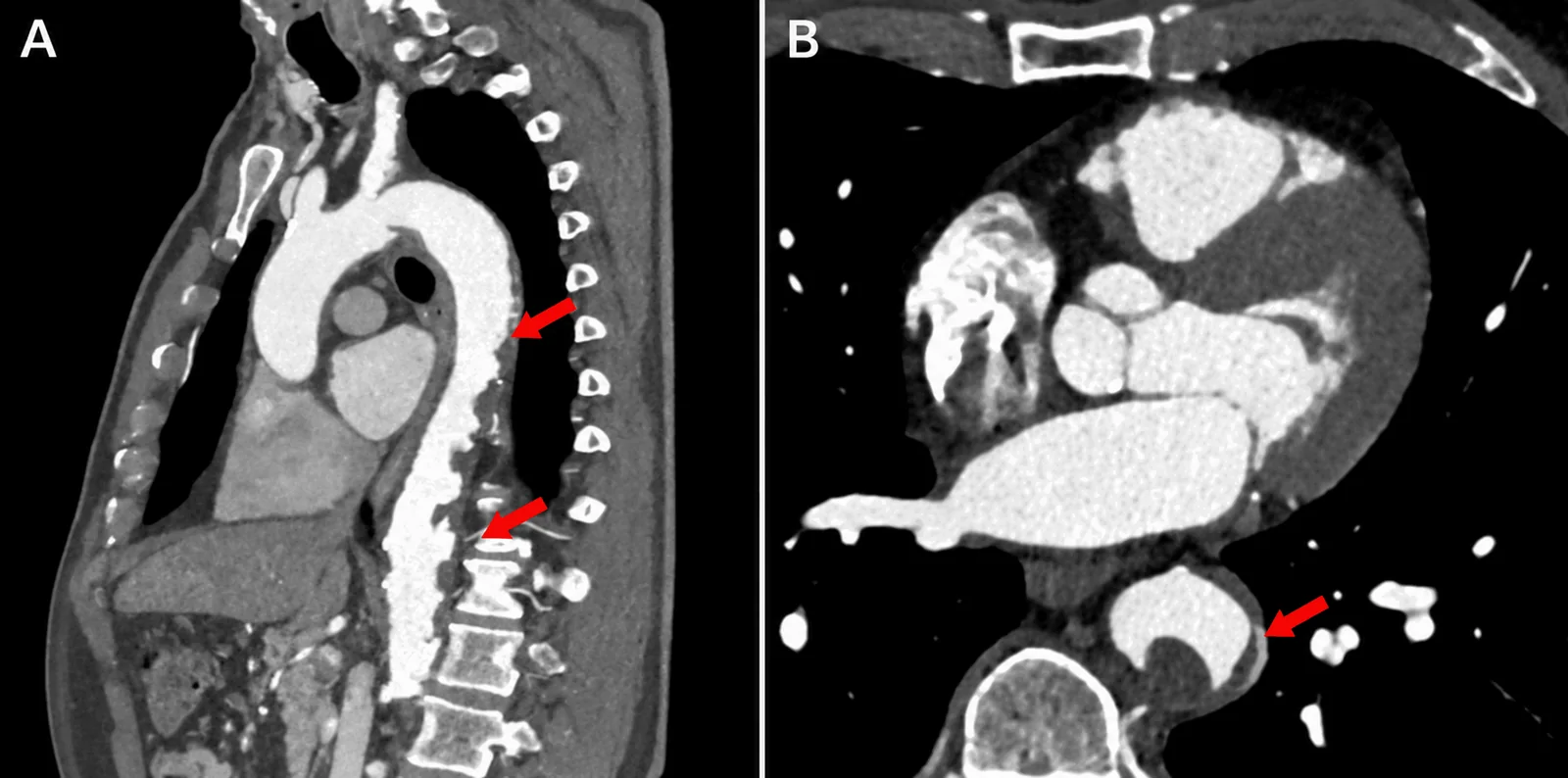
Preprocedural computed tomography angiography demonstrating extensive aortic atherosclerosis (A) Sagittal image demonstrating extensive irregular atherosclerotic plaque involving the thoracic and abdominal aorta with a “shaggy” appearance (arrows). (B) Axial image demonstrating bulky, eccentric, irregular atherosclerotic plaque involving the descending thoracic aortic wall (arrow).

Approximately one month after TAVR, he noted intermittent, painless violaceous discoloration of the first through third toes of the left foot that resolved spontaneously between episodes. Because the discoloration was painless, he did not seek medical attention.

On admission, he was hemodynamically stable with a blood pressure of 148/72 mmHg. For additional hemodynamic context, blood pressure around the time of TAVR was 138/81 mmHg. During the earlier AKI hospitalization, the lowest documented blood pressure was 128/72 mmHg, with no sustained hypotension or requirement for intravenous fluid resuscitation or vasopressor support. He reported fatigue and two days of vomiting but denied gross hematuria, chest pain, dyspnea, fever, rash, arthralgias, sinus symptoms, hemoptysis, or abdominal pain. He was incidentally found to have SARS-CoV-2 infection but was otherwise asymptomatic and managed supportively.

Examination demonstrated violaceous discoloration of the left first through third toes with palpable dorsalis pedis and posterior tibial pulses and intact capillary refill (Figure 2); no livedo reticularis, digital ulceration, or splinter hemorrhages were present. The right lower extremity had a prior above-knee amputation. The remainder of the examination, including fundoscopy for Hollenhorst plaques, was unremarkable.

**Figure 2:**
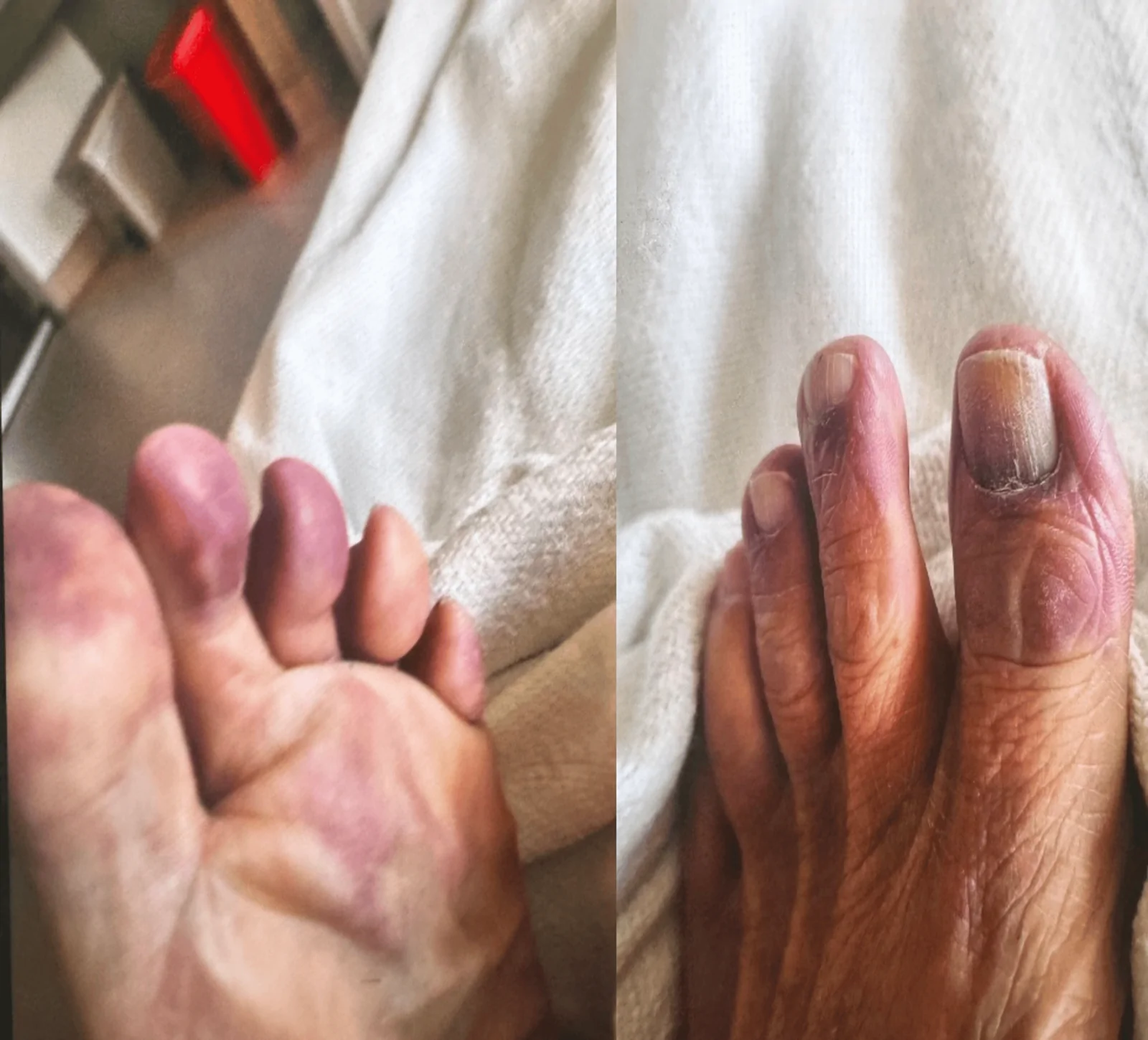
Clinical presentation of blue toe syndrome Clinical photographs of the left foot demonstrating violaceous discoloration of the first through third toes consistent with blue toe syndrome. Pedal pulses remained palpable despite distal digital ischemic change, a discrepancy characteristic of microvascular embolization rather than large-vessel occlusion.

Initial laboratory evaluation demonstrated AKI with microscopic hematuria, mild peripheral eosinophilia (absolute eosinophil count 0.64 ×10³/µL; 8.3%), elevated erythrocyte sedimentation rate and C-reactive protein, and sub-nephrotic proteinuria (Table 1). The eosinophil count was obtained before corticosteroid administration. Laboratory findings were not consistent with thrombotic microangiopathy: the platelet count was normal, and no schistocytes were identified on peripheral smear. Given the progressive renal dysfunction, hematuria, elevated inflammatory markers, eosinophilia, and acral discoloration, nephrology, rheumatology, and vascular surgery were consulted. Systemic vasculitis and rapidly progressive glomerulonephritis were the leading diagnostic considerations.

**Table 1:** Selected laboratory values on admission eGFR: estimated glomerular filtration rate; LDL: low-density lipoprotein; ESR: erythrocyte sedimentation rate; CRP: C-reactive protein; ANA: antinuclear antibody; ANCA: antineutrophil cytoplasmic antibodies; PR3: proteinase 3; MPO: myeloperoxidase; GBM: glomerular basement membrane; dsDNA: double stranded DNA

Laboratory Test	Patient Value	Reference Range
Serum creatinine	4.24 mg/dL	0.7–1.3 mg/dL
Blood urea nitrogen	59 mg/dL	7–20 mg/dL
eGFR	14 mL/min/1.73 m²	>60 mL/min/1.73 m²
Hemoglobin	10.6 g/dL	13.5–17.5 g/dL
White blood cell count	7.7 ×10³/µL	4.0–11.0 ×10³/µL
Platelet count	209 ×10³/µL	150–400 ×10³/µL
Peripheral smear	No schistocytes	—
Serum albumin	3.6 g/dL	3.5–5.0 g/dL
LDL cholesterol	80 mg/dL	<100 mg/dL
ESR	47 mm/hr	<20 mm/hr
CRP	4.136 mg/dL	<0.5 mg/dL
Absolute eosinophil count	0.64 ×10³/µL	0.0–0.50 ×10³/µL
Eosinophils	8.3%	0–6%
Complement C3	119.08 mg/dL	Normal
Complement C4	43.15 mg/dL	Normal
Urinalysis	1+ blood, trace protein, 3 RBC/HPF, 1 WBC/HPF	—
Urine protein-to-creatinine ratio	1.185 mg/mg	0.025–0.148 mg/mg
ANA	Negative	—
ANCA (PR3, MPO)	Negative	—
Anti-dsDNA	Negative	—
Anti-GBM	Negative	—
Hepatitis B/C, HIV, syphilis	Negative	—
Cryoglobulins	Negative	—
Serum protein electrophoresis/immunofixation	No monoclonal protein	—
Urine protein electrophoresis/immunofixation	Free monoclonal kappa band	—
Serum free light chain ratio	Normal	

Serologic evaluation included antinuclear antibody, antineutrophil cytoplasmic antibodies (ANCA) (with proteinase-3 and myeloperoxidase testing), anti-double-stranded DNA antibody, anti-glomerular basement membrane antibody, C3 and C4, hepatitis B and C serologies, HIV testing, serum and urine protein electrophoresis with immunofixation, serum free light chains, cryoglobulins, syphilis serology, and interferon-gamma release assay. The evaluation was unrevealing apart from elevated inflammatory markers, eosinophilia, and a free monoclonal kappa band on urine immunofixation. The serum free light chain ratio was normal, and no serum monoclonal protein was identified; these findings were considered insufficient to explain the clinical presentation. Complement C3 and C4 were normal, arguing against immune complex-mediated glomerular disease, although hypocomplementemia is itself an inconsistent finding in CES [5].

Urine microscopy demonstrated granular casts and dysmorphic erythrocytes, including acanthocytes. Given the progressive renal dysfunction, dysmorphic erythrocytes including acanthocytes, and concern for ANCA-negative pauci-immune vasculitis, empiric intravenous methylprednisolone (1 g daily for three days) was initiated after the relevant serologic studies had been collected but while results remained pending. Renal ultrasonography and non-contrast CT of the abdomen and pelvis excluded obstructive uropathy. CT-guided native kidney biopsy was performed two days after initiation of corticosteroid therapy.

Histopathologic examination demonstrated severe arteriosclerosis with biconvex, needle-shaped cholesterol clefts within intrarenal arteries and associated ischemic tubular and glomerular changes. Immunofluorescence and light microscopy showed no immune complex deposition, necrotizing lesions, or crescents (Figure 3). These findings established atheroembolic renal disease as the primary cause of his AKI.

**Figure 3:**
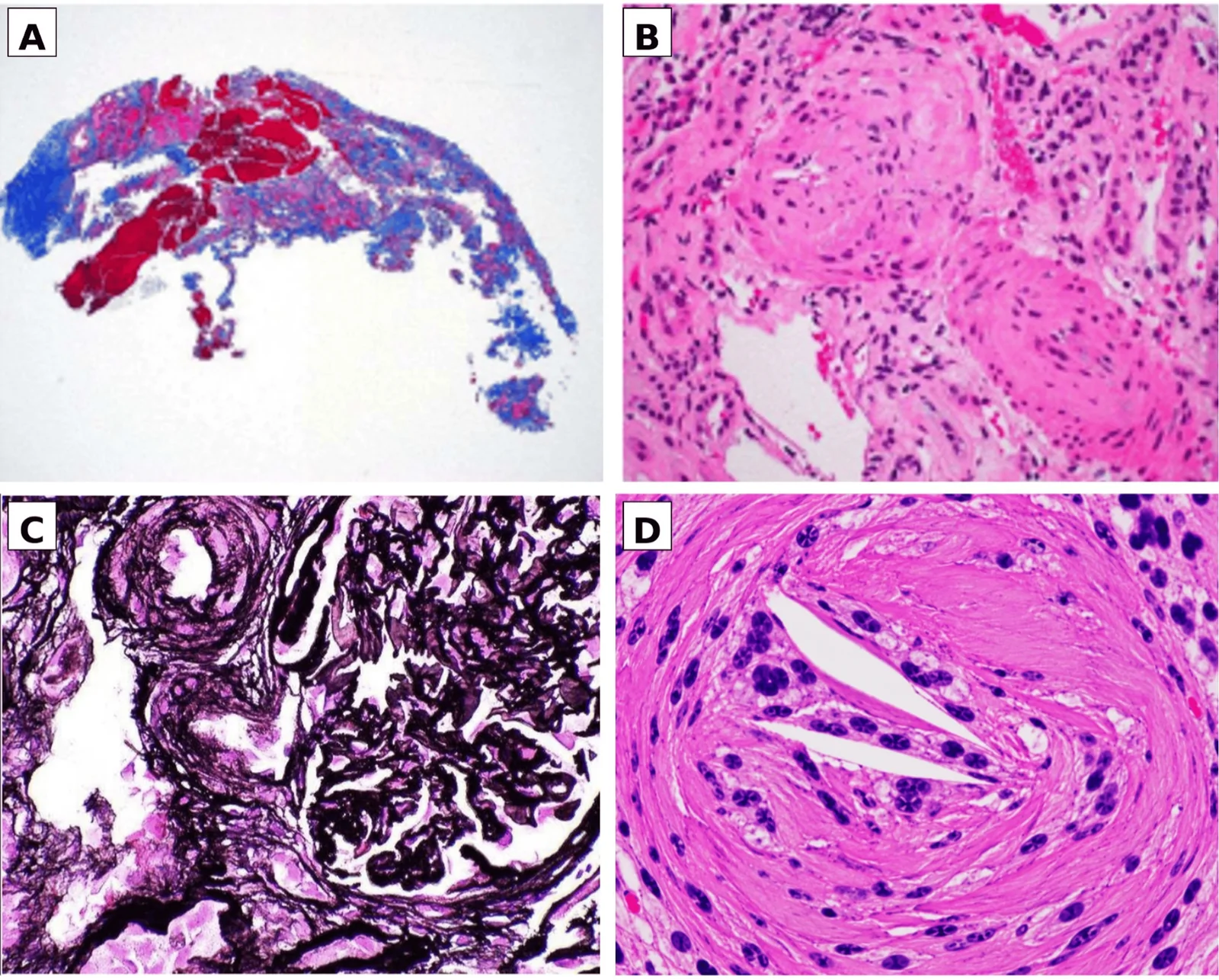
Kidney biopsy findings (A) Low-power view of renal cortex (Masson trichrome) demonstrating vascular and interstitial involvement. (B) Cholesterol clefts within an intrarenal artery with surrounding intimal reaction (hematoxylin and eosin). (C) Jones methenamine silver stain demonstrating ischemic glomerular changes without immune complex-mediated or crescentic glomerulonephritis. (D) High-power hematoxylin and eosin view demonstrating characteristic needle-shaped cholesterol clefts within an intrarenal artery with surrounding intimal reaction.

Corticosteroids were discontinued after the biopsy result. Losartan and empagliflozin were withheld, and aspirin was resumed after the biopsy. Vascular surgery documented an ankle-brachial index of 1.21 and toe-brachial index of 0.98 in the left lower extremity, arguing against large-vessel occlusive disease and supporting distal microembolization. Given the diffuse thoracoabdominal atherosclerosis, absence of a discrete resectable embolic source, and concern that further instrumentation could provoke additional embolization, no surgical or endovascular intervention was pursued. Skin biopsy was not performed because there was no suitable cutaneous lesion other than the ischemic digits themselves, and digital biopsy in a dysvascular limb carries a substantial risk of nonhealing. Conservative management with antiplatelet therapy, high-intensity statin therapy, blood pressure and glycemic optimization, avoidance of further arterial instrumentation, and close outpatient surveillance was advised.

Serum creatinine improved to approximately 3.1 mg/dL by discharge without the need for renal replacement therapy, and the toe discoloration remained stable without ulceration. He was discharged with nephrology, cardiology, and vascular surgery follow-up. At approximately three months after the AKI hospitalization, serum creatinine had improved further to 1.9 mg/dL without the need for renal replacement therapy, and the toe discoloration had improved. Table 2 summarizes the clinical timeline.

**Table 2:** Clinical Timeline AKI: acute kidney injury; CT: computed tomography; eGFR: estimated glomerular filtration rate; TAVR: transcatheter aortic valve replacement.

Time point	Clinical course
Day 0 (index TAVR)	Uncomplicated transfemoral TAVR with a 34-mm self-expanding valve. Preprocedural CT angiography showed extensive thoracic and abdominal aortic atherosclerosis with a "shaggy" appearance.
Day 1 post-TAVR	Serum creatinine 1.4 mg/dL, within the patient's preprocedural baseline range of 1.2-1.5 mg/dL.
~1 month post-TAVR	Intermittent, painless violaceous discoloration of the left first through third toes with spontaneous resolution between episodes.
~2 months post-TAVR	Routine outpatient testing showed serum creatinine 3.95 mg/dL (baseline 1.2–1.5 mg/dL).
~2.5 months post-TAVR (admission)	Repeat creatinine 4.24 mg/dL (eGFR 14 mL/min/1.73 m²); admitted for evaluation of severe AKI.
~2.5 months post-TAVR (hospital days 1–3)	Microscopic hematuria, peripheral eosinophilia (0.64 ×10³/µL), elevated inflammatory markers, and blue toe syndrome identified. Nephrology, rheumatology, and vascular surgery consulted.
~2.5 months post-TAVR (hospital days 3–5)	Autoimmune, infectious, and paraprotein studies collected; relevant serologic results remained pending when empiric intravenous methylprednisolone was initiated for suspected rapidly progressive glomerulonephritis. Urine microscopy demonstrated granular casts and acanthocytes.
~2.5 months post-TAVR (hospital days 5–7)	CT-guided kidney biopsy: severe arteriosclerosis with atheroembolic cholesterol clefts and ischemic changes, without immune-mediated glomerulonephritis.
~3 months post-TAVR (post-biopsy)	Corticosteroids discontinued; nephrotoxins withheld; aspirin resumed; high-intensity statin therapy and risk-factor modification instituted.
~3 months post-TAVR (discharge)	Creatinine improved to ~3.1 mg/dL; discharged with nephrology, cardiology, and vascular surgery follow-up.
~3 months after AKI hospitalization	Serum creatinine improved further to 1.9 mg/dL without renal replacement therapy; toe discoloration improved.

## Discussion

Several features of this case contributed to the initial diagnostic uncertainty. The presentation evolved over several weeks rather than immediately after the procedure, making contrast-associated nephropathy or progression of chronic kidney disease plausible initial explanations for the rising creatinine. At the same time, eosinophilia, elevated inflammatory markers, hematuria, and dysmorphic erythrocytes raised concern for ANCA-associated vasculitis. Because untreated rapidly progressive glomerulonephritis can result in irreversible renal injury, empiric corticosteroid therapy was initiated while tissue diagnosis was pending. The biopsy findings subsequently allowed prompt discontinuation of immunosuppression. More importantly, a subacute, stepwise decline in renal function over weeks is the most common renal presentation of CES; thus, the delayed course in this patient was characteristic rather than atypical [1,3].

Blue toe syndrome provided the pivotal clinical clue. Despite striking digital discoloration, pedal pulses were palpable, and the ankle-brachial and toe-brachial indices were normal, arguing against large-vessel occlusion. Preserved macrovascular perfusion despite distal digital ischemia is characteristic of cholesterol crystal microembolization and should prompt consideration of CES after recent arterial manipulation [2,5]. The differential diagnosis of blue toe syndrome remains broad and includes fibrinoplatelet microemboli from an upstream stenotic or occlusive iliofemoral lesion, antiphospholipid syndrome, cryoglobulinemia, thrombotic microangiopathy, vasculitis, and other systemic proinflammatory states. These alternatives were considered during this patient's evaluation. The normal toe-brachial index and absence of a focal proximal lesion argued against a fibrinoplatelet source amenable to angioplasty or bypass.

Preprocedural imaging in this patient demonstrated extensive aortic atherosclerosis with a classic shaggy-aorta appearance. Most directly relevant to this case, severe aortic wall thrombus on preprocedural multidetector CT was independently associated with ischemic stroke and periprocedural death in a prospective cohort of 641 patients undergoing transfemoral TAVR, with the association persisting across multivariable adjustment models [11]. A separate retrospective series identified aortic wall thrombus in 11.3% of transfemoral cases and found associations with acute renal failure (11.8% vs 3.2%), a sevenfold higher rate of disabling periprocedural stroke, and sixfold higher mortality in the shaggy-aorta subgroup [12]. Severe aortic atheroma has likewise been associated with increased periprocedural ischemic stroke and cardiovascular death and with worse two-year outcomes after TAVR [13].

Two considerations further support the relevance of these observations to TAVR. First, embolic debris generation during TAVR is near-universal and may be greater than historically appreciated. Self-expanding prostheses, as implanted in this patient, have been associated with a greater number of large particles than balloon-expandable devices in some filter analyses [9], although this signal is inconsistent across pooled analyses [8], and one shaggy-aorta series associated excess periprocedural stroke with balloon-expandable prostheses [12]. The clinical relevance of valve type to systemic, noncerebral atheroembolism therefore remains unresolved. Second, cerebral embolic protection devices are designed to reduce embolization to the cerebral circulation and, by design, do not protect the visceral, renal, or lower-extremity circulations; a full-body capture device demonstrated a median of 191 particles >150 µm per case [9,10]. Systemic cholesterol embolization after TAVR may therefore be underrecognized, particularly because delayed renal dysfunction is readily attributed to contrast-associated nephropathy or progression of chronic kidney disease and tissue confirmation is infrequently pursued in this population.

Definitive diagnosis in this case required kidney biopsy. Kidney tissue yields a positive diagnosis in more than 75% of patients with acute or subacute renal failure, while skin biopsy of the feet or lower legs has a comparable or superior reported yield approaching 92% and is less invasive; fundoscopy identifies Hollenhorst plaques in 10-25% of cases [1,3]. When the classic clinical triad is present, CES may be diagnosed without tissue confirmation [3]. In this patient, kidney biopsy also excluded a treatable glomerular disease that would have required ongoing immunosuppression. This illustrates the value of tissue diagnosis when management depends on distinguishing atheroembolism from immune-mediated renal disease [5].

The prognosis of atheroembolic renal disease is generally guarded. Across series, approximately 37-61% of patients require dialysis, with only a minority recovering sufficient renal function to discontinue it, and reported one-year mortality varies substantially by era and management intensity [1]. In the largest prospective cohort, independent predictors of dialysis and death included reduced baseline renal function, diabetes, heart failure, acute/subacute rather than chronic presentation, and gastrointestinal involvement [14]. Our patient had several adverse features, including preexisting chronic kidney disease, diabetes, long-standing hypertension, and a subacute presentation, underscoring the importance of risk-factor modification and close longitudinal follow-up.

Management of CES remains largely supportive and preventive. Further arterial instrumentation should be avoided when possible, and anticoagulation should be withdrawn when the risk-benefit balance permits because it is a recognized precipitant [1,3]. Statin therapy is commonly recommended for plaque stabilization and secondary cardiovascular prevention. Observational data have associated statin use with a reduced risk of end-stage renal disease (hazard ratio (HR) 0.02; P = 0.003) and improved survival, although these data do not establish causality [1,15]. Aggressive treatment of hypertension and heart failure and adequate nutritional support are additional components of supportive management. Although avoidance of systemic anticoagulation provides a theoretical rationale for peritoneal dialysis or heparin-minimized hemodialysis, available data show no clear difference in renal recovery or mortality by modality; the choice should therefore be individualized. Antiplatelet agents have not been shown to confer benefit in atheroembolic renal disease specifically [1], although antiplatelet therapy remained indicated in this patient for secondary cardiovascular prevention and post-TAVR care.

The role of corticosteroids in CES remains uncertain. Potential benefit has been reported in small series, particularly among patients with multisystem involvement, recurrent or progressive disease, and systemic inflammation; however, the largest prospective cohort found no improvement in renal or patient outcomes [14]. Recognition that cholesterol crystals activate the NLRP3 inflammasome with downstream IL-1β release has provided a mechanistic rationale for colchicine and IL-1 blockade in early CES, but clinical evidence supporting their use is lacking, and neither is guideline-endorsed for this indication [2,5]. Low-density lipoprotein (LDL) apheresis, iloprost, and pentoxifylline have been described in isolated reports without controlled evaluation [1].

This report has the limitations inherent to a single case. Although the temporal relationship to TAVR supports a procedure-related event, contributions from spontaneous atheroembolism, concurrent SARS-CoV-2 infection, volume depletion from vomiting, renin-angiotensin system blockade, or sodium-glucose co-transporter 2 (SGLT2) inhibition cannot be excluded. Skin biopsy was not performed for corroboration, and a brief course of high-dose corticosteroids preceded kidney biopsy and may have modified the histologic inflammatory infiltrate.

## Conclusions

CES is an uncommon but important potential complication after TAVR that can mimic systemic vasculitis and other causes of AKI. Progressive renal dysfunction accompanied by blue toe syndrome, eosinophilia, and elevated inflammatory markers weeks to months after arterial instrumentation should prompt consideration of CES, particularly when preprocedural imaging demonstrates extensive aortic atherosclerosis or a shaggy aorta. When the diagnosis remains uncertain, less invasive evaluation, including fundoscopy and skin biopsy when an appropriate lesion is available, may provide diagnostic evidence. Kidney biopsy is particularly valuable when immune-mediated glomerular disease remains a competing diagnosis because it can establish atheroembolism, prevent unnecessary immunosuppression, and redirect management toward supportive care and secondary cardiovascular prevention.
